# Chemokine ligand-receptor interactions as potential therapeutic targets for atopic dermatitis: from basic to clinical research

**DOI:** 10.3389/falgy.2026.1798037

**Published:** 2026-03-30

**Authors:** Rio Tsukamoto, Hsi-Hua Chi, Hiroki Ueno, Shiena Tanaka, Shoko Fujiyoshi, Sung-Il Lee, Masanori A. Murayama

**Affiliations:** Department of Animal Models for Human Diseases, Institute of Biomedical Science, Kansai Medical University, Osaka, Japan

**Keywords:** antibody, atopic dermatitis, chemokine, chemokine receptor, chemotaxis, leukocyte, migration

## Abstract

Atopic dermatitis (AD) is a chronic inflammatory skin disease that commonly causes eczema accompanied by severe itching on pathological skin lesions. Although the pathological mechanisms are not fully understood, epidermal barrier dysfunction and immune dysfunction are critical for the development of AD. Notably, skin-infiltrating immune cells play a crucial role in the development of atopic skin inflammation. Recent studies have demonstrated that the infiltration of inflammatory cells into skin lesion is regulated by various chemokine ligands-receptors interactions. In this review, we focused on the pathogenic role of chemokines and chemokine receptors in AD development. The lesional skin tissues of patients with AD highly express various chemokines to enhance the migration of immune cells via chemokine ligand-receptor interactions. Since thier the inhibition and blockade contribute to the regulation of inflammatory response in the lesional skins of AD, chemokine ligands and/or receptors are prospective targets for AD therapy. In fact, some blocking agents and antagonist have shown positive results in the improvement of the inflammatory phenotypes in AD model mice. Clinical trials are progressing slowly but steadily, suggesting that chemokine ligands-receptors interactions remain a prospective therapeutic target for AD.

## 1 Introduction

Chemotactic cytokine, chemokine ligand-receptor interactions contribute to the leucocyte recruitment in inflammatory tissues. At least 50 chemokines subtypes have been identified in mammals (1). Classical and major chemokines are all approximately 8–15 kDa in mass, and are commonly characterized by four conserved cysteine residues; intramolecular disulfide bonds typically join the first to the third, and the second to the fourth cysteine residues at the N-terminus. Based on the position of the first two conserved N-terminal cysteine residues, chemokines are categorized into four classes: CC-chemokines (β-chemokines), possessing two adjacent cysteine residues; CXC-chemokines (α-chemokines), possessing two cysteine residues separated by another amino acid (X); CX_3_C-chemokines (δ-chemokines), possessing two cysteine residues separated by three other amino acids (X_3_); and C-chemokines (γ-chemokines), with only one disulfide bridge between the second and fourth cysteine residues (2). Furthermore, CXC chemokines are further divided into the glutamate-leucine-arginine (ELR)-positive chemokines (CXCL1–3, and CXCL5–8), and ELR-negative chemokines (CXCL4, and CXCL9–11), based on the presence or absence of the three amino acid (Glu-Leu-Arg) motif before the first cysteine residue. Generally, ELR-positive chemokines promote angiogenesis, whereas ELR-negative chemokines are IFN-inducible and inhibits angiogenesis (3). In terms of chemotaxis, ELR-positive chemokines are mostly chemotactic for neutrophils, while ELR-negative chemokines act on natural killer (NK) cells and activated T cells (4). Negatively charged glycosaminoglycans control the binding of chemokines (5), and some chemokines are processed by matrix metalloproteinase (MMP)-mediated proteolysis (6). Recently, a family of fifth chemokine, the chemokine-like factor (CKLF) family, has been identified (we define these as *ε*-chemokines in this review). This chemokine family has different structural features compared to typical chemokines (7). CKLF1 has a contiguous CC-chemokine structure similar to CCL17 and CCL22, but lacks the additional C-terminal cysteine residues (8).

Various immune cells express chemokine receptors on their cell surfaces. Classical chemokine receptors are G-protein-coupled transmembrane receptors (GPCRs). In addition to classical chemokine receptors, atypical chemokine receptors (ACKRs) have emerged as critical regulators of inflammatory responses. ACKRs bind chemokines without inducing conventional G protein–mediated signaling; instead, they modulate chemokine availability and gradients, thereby fine-tuning immune cell recruitment (9). The chemokine ligand-receptor interactions are summarized in Table 1.

**Table 1:** The chemokines and chemokine receptors (classical and atypical).

Name	Other names	Receptors (classical)	Receptors (atypical)
CXC chemokine (α chemokine)			
CXCL1	Gro-α, GRO1, NAP-3	CXCR2	ACKR1
CXCL2	Gro-β, GRO2, MIP-2α	CXCR2	ACKR1
CXCL3	Gro-γ, GRO3, MIP-2β	CXCR2	ACKR1
CXCL4	PF-4	CCR1, CXCR3	ACKR1
CXCL4L1	PF4V1	CXCR3	
CXCL5	ENA-78	CXCR1, CXCR2	ACKR1
CXCL6	GCP-2	CXCR1, CXCR2	ACKR1
CXCL7	NAP-2, CTAPIII, β-Ta, PEP	CXCR2	ACKR1
CXCL8	IL-8, NAP-1, MDNCF, GCP-1	CXCR1, CXCR2	ACKR1
CXCL9	MIG, CRG-10	CXCR3	
CXCL10	IP-10, CRG-2	CXCR3	
CXCL11	I-TAC, β-R1, IP-9	CXCR3	ACKR1, ACKR3, ACKR4
CXCL12	SDF-1, PBSF	CXCR4	ACKR3
CXCL13	BCA-1, BLC	CXCR5	ACKR1, ACKR4
CXCL14	BRAK, bolekine	Unknown	
CXCL15	Lungkine, WECHE	Unknown	
CXCL16	SRPSOX	CXCR6	
CXCL17	DMC, VCC-1	Unknown	
CC chemokine (β chemokine)			
CCL1	I-309, TCA3	CCR8	
CCL2	MCP-1	CCR1, CCR2, CCR4, CCR5	ACKR1, ACKR2
CCL3	MIP-1α	CCR1, CCR3, CCR5	ACKR2
CCL3L1	LD78β	CCR1, CCR3, CCR5	ACKR2
CCL4	MIP-1β	CCR5	ACKR2
CCL4L1	LAG-1	CCR5	
CCL5	RANTES	CCR1, CCR3, CCR4, CCR5	ACKR1, ACKR2
CCL6	C-10, MRP-1	CCR1	
CCL7	MARC, MCP-3	CCR2, CCR3	ACKR1, ACKR2
CCL8	MCP-2	Human: CCR1, CCR2, CCR3, CCR5, Mouse: CCR8	Human: ACKR1, ACKR2 Mouse: ACKR1, ACKR2
CCL9/10	MIP-1γ, MRP-2, CCF18	CCR1	
CCL11	Eotaxin-1	CCR3	ACKR2
CCL12	MCP-5	CCR2	
CCL13	MCP-4, NCC-1, Ckβ10	CCR2, CCR3, CCR5	ACKR1, ACKR2
CCL14	HCC-1, MCIF, Ckβ1, NCC-2, CCL	CCR1	ACKR1, ACKR2
CCL15	Leukotactun-1, HCC-2, MIP-5, NCC-3	CCR1, CCR3	
CCL16	HCC-4, NCC-4, LEC (human only)	CCR1, CCR2, CCR5	ACKR1
CCL17	TARC, dendrokine, ABCD-2	CCR4	ACKR1, ACKR2
CCL18	PARC, DC-CK1, AMAC-1, Ckβ7, MIP-4	CCR8	ACKR6
CCL19	MIP-3β, ELC, Exodus-3, Ckβ11	CCR7	ACKR4, ACKR5
CCL20	MIP-3α, LARC, Exodus-1, Ckβ4	CCR6	
CCL21	SLC, 6Ckine, Exodus-2, Ckβ9, TCA-4	CCR6, CCR7	ACKR4
CCL22	MDC, DC/β-CK	CCR4	ACKR1, ACKR2
CCL23	MPIF-1, Ckβ8, MIP-3, MPIF-1	CCR1	
CCL24	Eotaxin-2, MPIF-2, Ckβ6	CCR3	
CCL25	TECK, Ckβ15	CCR9	ACKR4
CCL26	Eotaxin-3, MIP-4α, IMAC, TSC-1	CCR3, CX3CR1	
CCL27	CTACK, ILC, Eskine, PESKY, skinkine	CCR10	
CCL28	MEC	CCR3, CCR10	
C chemokine (γ chemokine)			
XCL1	Lymphotactin α, SCM-1α, ATAC	XCR1	
XCL2	Lymphotactin β, SCM-1β	XCR1	
CX3C chemokine (δ chemokine)			
CX3CL1	Fractalkine, Neurotactin, ABCD-3	CX3CR1	
CKLF (ε chemokines)			
CKLF1		CCR3, CCR4, CCR5	

In this review, we summarize the pathogenic roles of chemokine ligands and receptors in atopic dermatitis (AD). Furthermore, we provide important evidence regarding the potential clinical benefits of chemokine inhibition in patients with AD.

## 2 Atopic dermatitis

AD is one of the most common and chronic inflammatory skin diseases, characterized by pruritic, erythematous, and edematous lesions, with approximately 15%–20% of children and 1%–3% of adults affected worldwide. AD can occur at any age, with approximately 60% of patients developing the disease within the first year of life (10).

Epidemiological studies have shown that AD is more prevalent in industrialized regions, particularly in Northern Europe, North America, and parts of East Asia, whereas lower prevalence rates are reported in many low- and middle-income countries. However, recent data suggest a rising incidence of AD in rapidly urbanizing regions, indicating that environmental and lifestyle factors play a substantial role in AD development (11). The underlying immune dysfunctions in AD is characterized by a type 2 helper T cell (Th2)-shifted abnormal immune response. This immune dysregulation coordinates with the skin barrier impairments and pruritus.

The development of AD begins with epidermal barrier impairment, often due to *filaggrin* mutations or inflammation-induced barrier dysfunction (12). Barrier disruption promotes the release of epithelial-derived alarmins such as thymic stromal lymphopoietin (TSLP), IL-33, and IL-25, which are contributes to leading to type-2 immune polarization and are useful for biomarkers in endophenotypic profiling of AD (13). In fact, TSLP and IL-25 activate DCs, and also IL-25 and IL-33 activates ILC2s to express OX40L. Ligation of OX40L/OX40 contributes the differentiation of Th2 cells (14–18). The infiltrated Th2 cells into inflamed skin of patients with AD produces type 2 cytokines such as IL-4, IL-5, IL-13, and IL-31 contributes barrier dysfunction, IgG production, and eosinophil infiltration (19).

The resultant IL-4, IL-5, and IL-13 production further damages barrier integrity and enhances the release of chemokines from keratinocytes, establishing a vicious cycle of inflammation and itching (20). Keratinocytes, as the first line of defense, play a pivotal role not only in forming the physical barrier but also in sensing environmental insults. DCs, particularly the inflammatory and Langerhans-type subsets (LCs), bridge the innate and adaptive immune systems by presenting antigens and producing a variety of cytokines and chemokines that reinforce type-2 immune polarization. Th2 cells, once activated, secrete IL-4, IL-5, and IL-13, amplifying inflammation, impairing epidermal barrier function, and stimulating keratinocytes to produce further inflammatory mediators. Eosinophils, mast cells, and basophils infiltrate the dermis, releasing mediators such as histamine, major basic protein, and IL-31, which contribute to pruritus and sustained inflammation (21, 22). The recruitment and spatial organization of these immune cells within lesional skin are tightly regulated by a complex network of chemokines. Therefore, the regulation of chemokine ligand-receptor interaction is important for AD therapy.

## 3 Chemokines in AD pathophysiology

Various chemokines are highly expressed in the serum and inflamed skin of patients with AD. For instance, CC-chemokines CCL2, CCL4, CCL5, CCL11, CCL13, CCL17, CCL18, CCL22, CCL26, CCL28, CXC-chemokines CXCL9–11, and CLFK1 are highly expressed in the serum of patients with AD (23–34). Moreover, CCL1, CCL3, CCL5, CCL17, CCL18, CCL20, CCL23, CCL24, CCL27, CCL28, CXCL1, CXCL5, CXCL8, CXCL12, and CLFK1 are increased in the lesional skins of patients with AD compared to non-lesional skins of patients with AD or intact skins of healthy individuals (25, 33, 35–42). However, chemokines related to Th1 response (CCL28 and CXCL-9–11) are decreased in blood of patients with pediatric AD (43). Specifically, CCL5, CCL7, and CCL24 are increased in allergen-challenged skin sites in patients with AD (44). The mRNA expression of *CCL2*–*4*, *CCL7*, *CCL11*, *CCL13*, CCL17*, CCL18*, *CCL22*, CCL24*, CCL26*, *CXCL8–11*, and *CKLF1* are increased in skin of patients with AD (33, 38, 45–54). In addition, *CCL1–5*, *CCL7*, *CCL8*, *CCL11*, *CCL13*, *CCL17*, *CCL18*, *CCL20*, *CCL22*, and *CCL27* mRNA are increased in epidermis of patients with AD during atopy patch test (55). Interestingly, the expression of CCL5*, CCL17*, and *CCL22* mRNA in lesional skin shows distinct profiles between adult-onset and pediatric onset AD (56). In OVA-induced AD model mice, *Ccl1*, *Ccl3*, *Ccl4*, *Ccl8*, *Ccl11*, *Ccl17*, *Ccl22*, *Ccl24*, *Cxcl9* and *Cxcl10* mRNA levels are increased, whereas *Ccl17* mRNA expression are decreased in the skin (57–59). In other AD model mice, *Ccl11*, *Cxcl1*, *Cxcl10*, and *Cxcl12* mRNA expression are increased in skin (60).

 Peripheral blood mononuclear cells (PBMCs) of patients with AD highly express CCL2, CCL3, CCL4, and CCL5 (23). Furthermore, *CCL3*, *CCL17* and *CXCL8* mRNA expression are increased, whereas *CCL8* and *CCL13* mRNA expression are decreased. Additionally, TLR2 ligand enhances the mRNA expression of *CCL5*, *CCL8*, *CCL13*, *CCL18*, and *CCL21* in PBMCs of patients with AD (61, 62). Various immune cells produce different chemokines in the lesional skin of patients with AD: DCs and LCs produce CCL1, CCL3–5, CCL17, CCL18, and CCL22 (36, 63–67); macrophages produce CCL13 and CCL18 (68, 69); mast cells produce CCL1, CCL3, CCL4, and CXCL8 (36, 70); and basophils produce CCL3 and CXCL12 (71). Platelets highly express CCL17 in patients with AD (27).

Other skin-tissue components also produce chemokines; keratinocytes produce CCL26 (48); fibroblasts produce CCL11, CCL19, CCL24, CCL26, and CXCL12 (28, 48, 72); and endothelial cells produces CX_3_CL1 in patients with AD (73).

Furthermore, the expression of these chemokine is induced by allergens and AD-related inflammatory mediators. For example, *S. aureus* infection enhances CCL7 production from LCs (74), CXCL8 release from mast cells (75), and inhibits Th1 cell-recruiting chemokines (CXCL9 and CXCL11) production in PBMCs (76). *S*. *pseudintermedius*-activated TLR2 triggers CCL5 production from canine keratinocytes (77). Staphylococcal products enhance CCL18 expression from DCs and LCs (65), but reduces CXCL10 expression in macrophages from patients with AD (78). Regarding house dust mites, extracts from *D*. *pteronissinus* increase *CCL2* and *CCL8* mRNA expression in monocytes (79), and increase *CXCL8* mRNA from bronchial epithelial cells, but not keratinocytes (80). Mite antigens from *D. farinae* induces CCL17 expression in keratinocytes (81). Some fungi increase CCL22 production but reduces CXCL10 production from PBMCs of patients with AD (82). In contrast, antimycotics suppress CCL2, CCL5, and CCL27 expression in human keratinocytes (83), and suppress CCL17 and CCL22 expression in PBMCs from patients with AD (84). Immunotherapy using house dust mite allergens in patients with AD decreases serum CCL17 and CCL22 levels, which correlates with reductions in disease severity (85).

Histamine can increase *CCL3* and *CCL4* mRNA levels, and CCL3 at the protein level via the histamine H4 receptor from NK cells (86), as well as CCL18 expression in M2 macrophages (87). PGE2 inhibits CCL27 production from keratinocytes (88). Various cytokines such as IL-4, IL-13, IFN-*γ* and TNF-α induce CCL2, CCL5, CCL26, and CCL27 expression in keratinocytes (88–93). Moreover, IL-29 induces the expression of CXCR3 ligands, CXCL9–11 in keratinocytes (94, 95). IL-31 induces CCL2, CCL18, CXCL1, and CXCL8 from keratinocytes and eosinophils (95). TNF-α and IL-1β produces the expression of CCR10 ligands, CCL27 and CCL28 from keratinocytes via different signaling pathways (96, 97). TNF-α and IFN-γ increase CCL2, CCL5, CCL17, CCL20, CCL22, CCL28, and CXCL8 production (98–101) as well as IL-33 production from keratinocytes (102). IL-33 induces CXCL8 expression from mast cells (103). IL-4 induces CCL17 expression in macrophages (104). IL-4- and CD40-activated B cells produce CCL17 and CCL22 (105). TNF-α and IL-4 increase CCL17 production from LCs (106). CCL17 augments TNF-α-induced CCL27 production in keratinocytes (107). TNF-α induces CCL11 and CXCL8 secretion from dermal fibroblasts (108, 109). Cross-talk between TNF-α and IFN-γ is essential for CXCL10 production in monocytes (110); however, TNF-α, but not IFN-γ, is the main inducer of CXCL10 in skin fibroblasts from patients with AD (111). Anti-IL-4Rα, anti-IL-13, and anti-IL-13Rα therapy in patients of AD decreased CCL13, CCL17, CCL18, and CCL22 expression in skin (112–115). JAK/SYK inhibitor therapy in patients with AD decreases CCL13, CCL17, CCL18, CCL20, CCL22, CXCL9, and CXCL11 expression in the skin (116). JAK1 inhibitor therapy in patients with AD decreases CCL17 expression in skin of patients with AD (117).

It is reported that *CCL2*, *CCL3*, *CCL5*, *CCL11*, and *CCL22* gene polymorphisms are associated with the development of AD (118–124). CCL13, CCL17, CCL22, CCL26–28, and CX_3_CL1, but not CCL11 and CCL24 expression levels in the serum correlates with the severity of patients with AD (24, 26, 27, 32, 42, 73, 112, 125–127). CCL3 levels correlate with the infiltration of neutrophils, basophils, mast cells, and macrophages into allergen challenged skin of patients with AD (128). CCL5 levels are associated with IgE levels, LDH levels, and eosinophils counts in the serum of patients with AD (23). CCL11 and CCL28 expression levels are associated with the infiltration of eosinophils in the lesional skin of patients with AD (42, 51, 52). CCL13 levels correlates with the number of resident macrophages in skin lesion of patients with AD (51).

The imbalance of type 1 helper T cell (Th1)- and Th2-prone chemokines is associated with the pathogenesis of AD (129, 130). Th2 polarization such as CCL17 exists in infants and children, while Th1 polarization such as CXCL9–11, is detected in adults with AD (131). Interestingly, Th1-prone chemokines are decreased in blood of patients with pediatric AD (132). Th2 chemokines, CCL17, CCL22, and CCL27, are important markers of AD severity during childhood (133), whereas Th1 chemokines, CCL28, and CXCL9–11 are inversely correlated with the disease activity of patients with pediatric AD (43). Increased levels of CCR4 ligands, CCL17 and CCL22 correlate with blood eosinophil counts, IgE levels, and the severity AD during childhood (134). CCL17 has emerged as the most reliable indicator of disease severity across all age groups (135). CCL17 levels in the skin implicated in the development of AD during childhood (136, 137), and correlates with disease severity of AD in pediatric (138–140) and adult patients (141, 142). Furthermore, CCL17 levels in umbilical cord serum predict the development of AD in infancy (143). CCL17 and CCL25 levels in the serum correlate with the severity of food-sensitized infants with AD (144). CCL17 levels correlate with CCL27 levels in the serum of patients with AD (145). Serum CCL27 levels correlated various clinical parameters of AD in childhood (146). Cord blood CCL22 levels are associated with total IgE levels during preschool age (147). CCL22 produced by monocyte-derived DCs reflects disease activity in patients with AD (148, 149). The CCL22/CXCL10 ratios in cord blood is associated with the allergic sensitization in early childhood (147, 150). In addition, serum CX_3_CL1 concentration correlates with IgE, and IL-31 levels, and severity in pediatric patients with AD (151).

## 4 Chemokine receptors in AD development

**Figure 1:**
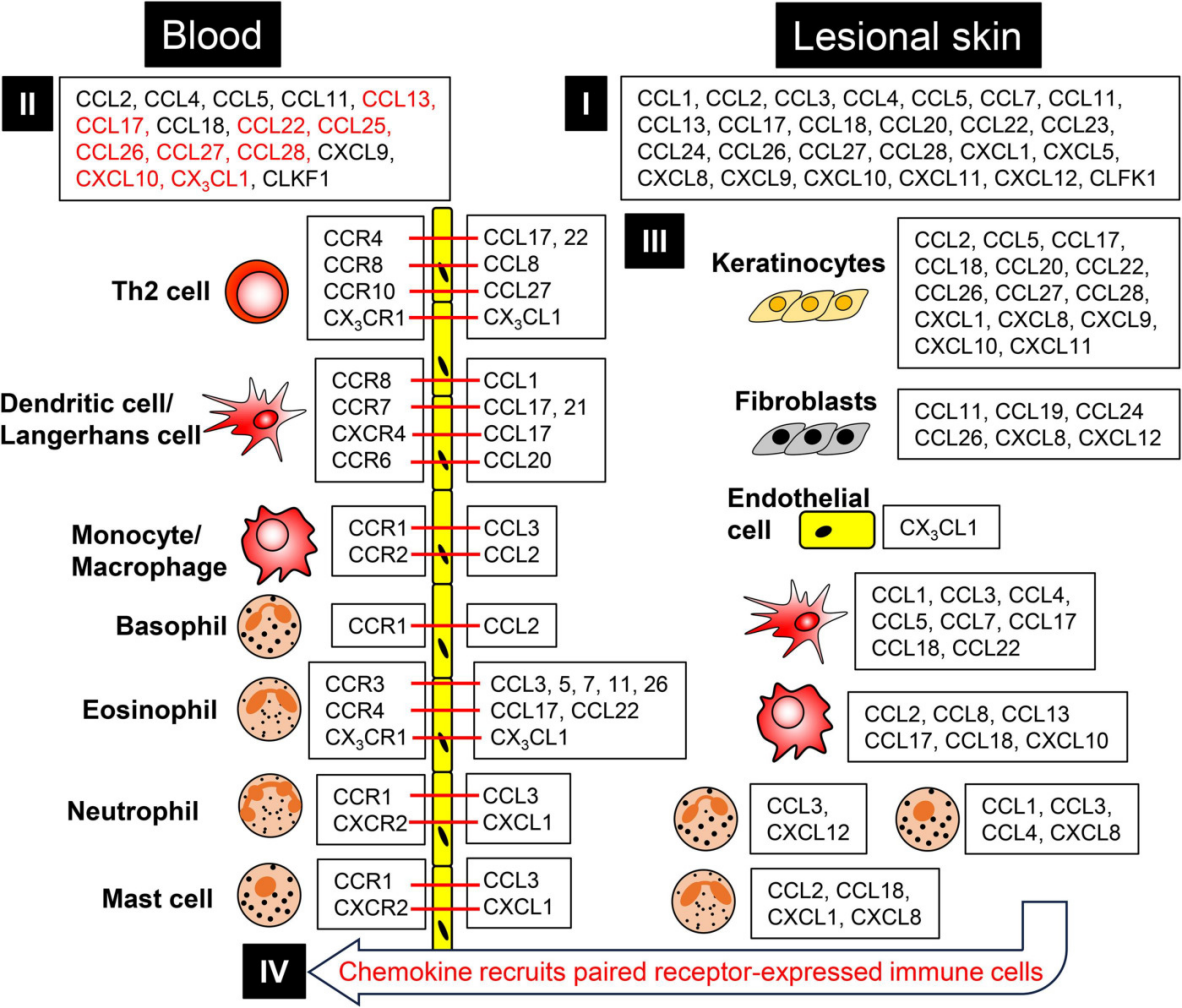
The major contribution of chemokine ligand-receptor interactions in AD. **(I,II)** Various chemokines are highly expressed in peripheral blood **(I)** and lesional skin (**II**) in patients with AD. Some chemokine (indicated as characters in red) levels are correlated with the severity of patients with AD. **(III)** The major source of chemokines at lesional skin on patients with AD. **(IV)** Chemokine recruits paired receptor-expressed immune cells. Red bar shows chemokine ligand-receptor interactions.

In contrast to chemokine, susceptible gene polymorphisms for chemokine receptors in AD development have not beed identified (152, 153). However, various chemokine receptors contribute to AD pathogenesis, similar to chemokines (Figure 1). CCR1 is expressed on infiltrated neutrophils, basophils, mast cells, and macrophages in allergen-challenged skin of patients with AD (128), as well as on DCs in patients with AD (55). CCR2 is expressed in monocytes, macrophages and DCs in the inflamed skin of patients with AD (55, 154). CCR3 is expressed in eosinophils in the skin and peripheral blood of AD model mice (155), but is not detected in CD4^+^ T cells in the peripheral blood of patients with AD (156). CCR3 expression levels are associated with the infiltration of eosinophils in lesional skin of patients with AD (44, 52). Animal experiments show that CCR3 is essential for the infiltration of eosinophils into the inflamed skin of AD; however, CCR3 deficientcy does not influence the infiltration of neutrophils and lymphocytes into the inflamed skin of AD model mice (157). CCR3 is also expressed in fibroblasts and keratinocytes in the lesional skin of patients with AD (48, 158), and TNF-α induces CCR3 expression in fibroblasts (109). Staphylococcal enterotoxin B induces CCR4 expression in PBMCs of patients with AD (159). Percutaneous application of component from *S. aureus* induces CCR4^+^ cells into mouse skin via CCL17 from epidermal cells, and the infiltration is inhibited by anti-CCL17 antibody (160). In patients with AD, CCR4^+^ T cells are infiltrated into inflamed skin (161, 162), and CCR4-expressing Th2 cells are increased in the skin lesions of AD model mice (58). CCR4^+^ Th2 cells are increased in the peripheral blood of patients with AD among adult and children (162–166), and the CCR4 expression levels on PBMCs reflects disease activity of AD (167). Furthermore, CCR4 and also CCR5 are expressed in regulatory T cells in the peripheral blood of patients with AD (168). And CCR5 is expressed in Langerin-negative CD1a^+^ DCs in the skin of patients with AD (55). Immunostimulatory sequence CpG injection elicited the infiltration of CCR5^+^ cells into the skin of AD model mice (169). Comprehensive bioinformatics analysis and machine learning showed that CCR5 expression correlates with Th1, Th2, and IL-17-producing helper T cells (Th17 cells) in keloid with AD (170). CCR5 and CXCR3 are selectively expressed on Th1 cells, the decrease in these expression in CD4^+^ T cells associates disease activity of patients with AD (156). CCR6 is expressed on the infiltrated DCs and T cells in the skin of patients with AD (101). The skin commensal Yeast, *Malassezia* spp. triggers the recruitment of CCR6^+^ memory Th17 cells into the skin of patients with AD (171). Langerin-negative CD1a^+^ DCs also express CCR6 in the skin of patients with AD (55). In contrast, IFN-*γ* expressing CCR6^+^ T cell are decreased in the PBMCs of patients with AD (172). Comprehensive bioinformatic analysis showed that CCR7 expression levels correlates with the number of naïve CD4^+^ T cells in patients with AD (173). DCs and T cells express CCR7 in the skin of patients with AD (28, 174). Furthermore, TNF-α and IFN-*γ* induce CCR7 expression in keratinocytes (175). CCR8 is expressed in LCs, monocytes, and T cells of patients with AD (36). CXCR3 is mainly expressed on Th1 cells of patients with AD, but the population is at the same levels as healthy controls (34). The resident natural killer T (NKT) cells uniquely express CXCR4 in inflamed skin of patients with AD (72), and CXCR4 is also expressed in LCs in AD model mice (176). CXCR5 is expressed in T follicular helper (Tfh) cells in the lesional skin of patients with AD (177). During childhood, CXCR5^+^ Tfh cells in PBMCs are increased in extrinsic AD, but not intrinsic AD (178). The expansion of CXCR5^+^ Tfh cells is associated with disease severity in AD during childhood (179). CX_3_CR1 is expressed in the skin of patients with AD; however, the populations of CX_3_CR1^+^ CD8^+^ T cells and CD14^+^ monocytes in PBMCs are decreased, while CX3CR1^+^ CD4^+^ T cells and CD16^+^ NK cells are not (73).

## 5 The role of chemokine ligands-receptors in AD development

Chemokines and their corresponding receptors contribute to the recruitment of lymphocytes into the lesional skin of patients with AD and animal models (Figure 1). CCR2 KO mice develop severe AD-like inflammation due to decrease in Ly6C^hi^ monocytes infiltration into the skin (180). CCL2; a ligand for CCR2, CCR4 and CCR5, and CCL3; a ligand for CCR1 and CCR5, induce the recruitment of T cells, macrophages, neutrophils, and eosinophils into the skin of patients with AD. Furthermore CCL2, but not CCL3 induces the recruitment of basophils into the skin of patients with AD (181). CCL2, but not CCL5(a ligand for CCR1, CCR3–5) from IL-13-stimulated keratinocytes enhances the chemotactic activity of CCR4^+^ T cells from patients with AD (182). CCL5 from LCs contributes the infiltration of Th1 cells and eosinophils into the skin in AD (183).

CCR3-deficient mice showed an atopic phenotype at comparable levels with controls in AD model mice, along with a decrease in the infiltration of eosinophils in the skin (157). The CCL7/CCR3 axis recruits eosinophils to the inflamed skin of AD model mice following *S. aureus* exposure (184) and contributes to pruritic signaling in Schwann cells in AD (49). CCL11, a ligand for CCR3 from dermal fibroblasts and endothelial cells migrates eosinophils into the lesional skin in patients with AD (185, 186). The CCL26/CCR3 axis on fibroblasts contributes to tissue remodeling in AD (48). CCL26/CCR3 and CX_3_CL1/CX_3_CR1 axis may recruit NK cells and eosinophils into the skin in AD (187).

CCR4 deficiency reduces disease severity in AD model mice, accompanied by a decrease in the infiltration of mast cells, eosinophils and Th2 cells into the inflamed skin (58). CCL17 transgenic mice show a severe phenotype in AD models with an increase in the Th2-type response (188). The CCL22/CCR4 axis recruits memory Th2 cells into the skin (189). CCL17 expressed from keratinocytes migrate CCR4^+^ Th2 cells into the lesional skin of patients with AD (190, 191). CCL17 is also required for CCR7- and CXCR4-dependent migration of DCs in skin of AD model mice (176). And the CCL21/CCR7 axis contributes the infiltration of DCs into lesional skin of patients with AD (192). The CCL19/CCR7 axis contributes to TNF-α and IFN-γ induced skin inflammatory responses in keratinocytes (175).

CCR8 KO, and CCL8/CCL12 double KO mice, but not CCL12 KO mice have decreased skin inflammation in the OVA-induced AD model (193). CCL1 synergizes with CXCL12 for the recruitment of CCR8^+^ T cells and DC subsets into atopic skin (36). The CCL8/CCR8 axis promotes the recruitment of Th2 cells into allergen-inflamed skin in OVA-induced AD model mice (193). CCL18, a ligand for CCR8 recruits memory T cells into the skin of AD model mice (69).

The CXCR3 ligands, CXCL9–11, are highly expressed in the inflamed skin of patients with AD; however, the population of CXCR3-expressing Th1 cells in the skin at the same levels in patients with AD and healthy controls (34). Although the CXCL9–11/CXCR3 axes may contribute the Th1 cell recruitment into the lesional skin of patients with AD, the population of CXCR3-expressing T cells is not important for the disease severity of AD (167).

Other chemokine ligands/receptors are also contributed to skin inflammation. CCL27/CCR10 axis is an essential chemokine for memory T cell homing into the inflamed skin in AD (96). CXCL12 mainly from skin fibroblasts enriches CXCR4^+^ NKT cells clusters in skin lesions of the AD model (72). CX_3_CL1/CX_3_CR1 axis regulates Th1 and Th2 cell retention in the inflamed skin of patients with AD. CX_3_CR1 KO mice develop neither skin lesions nor lung inflammation in AD model (194).

## 6 The contribution of chemokine ligands-receptors for therapeutic target in AD

The classical chemokine receptors, which is GPCRs regulate multiple downstream signaling cascades, such as the JAK-STAT pathway (195). JAK inhibitors have already used for treatment of AD (196). Furthermore, the blockades of Th2-prone cytokines (IL-4, IL-13, and IL-31) are available as clinical agents for AD (197). According to clinical reports at phase 3 trials, the over half of JAK1-targeted upadacitinib-treated patients, and IL-4 and IL-13-targeted dupilumab-treated patients with AD showed treatment-emergent adverse events (TEAE), and the nearly half of IL-31-targeted nemolizumab-treated patients with AD showed TEAE at 16 weeks from treatment (198, 199). Thus, other clinical options need to be considered. In animal experiments, anti-CCL27 improves skin inflammation in AD model mice by neutralizing the CCL27-CCR10 interaction (96, 200). A CXCR2 antagonist, SB225022 ameliorates AD-like skin lesions by reducing mast cell recruitment and IL-17A production in AD model mice (201). An anti-CXCR2 antibody also improves inflammatory phenotypes in AD model mice (202). The endogenous CXCR4 antagonist prevents the allergic response in AD model mice (203). A CX_3_CL1 antagonist reduces features of AD in AD model mice (194).

CCL17 and CCL22 are ligands for CCR4. It has already been reported that anti-CCL17 and anti-CCL22 antibodies, and a CCR4 antagonist ameliorated the severity of AD model mice (204). The CCR4 antagonist, Compound 22 ameliorates skin lesions in AD model mice with reduction in Th2 cells, Th17 cells, and IgE levels (58, 205, 206). For clinical application, the oral CCR4 antagonist PPT193 showed clinical improvement in subjects with AD in a Phase 1 clinical trial (207). Furthermore, the neutraligand of CCL17, GPN279 showed therapeutic effect for patients with AD in the randomized double-blind placebo-controlled cosmetic trial (208).

Recent research into the various immune profiles and molecular signatures of AD has showed distinct endotypes such as extrinsic/intrinsic subtype, difference in age, and geographic variations (209). As a representative example, Th1-, and Th2-prone chemokine profiles are different between AD endotypes on adult and childhood (131, 132). CCL17 and Th17-prone chemokine, CCL22 are increased in extrinsic AD and intrinsic AD, but CCL17 levels were significantly higher in extrinsic than intrinsic AD (210, 211). Thus, chemokine-targeted clinical trials are required for consideration of the difference of endotype.

## 7 Conclusion

This review collates and summarizes current advances in the understandings of chemokine ligands-receptors in the pathogenesis of AD. These studies suggest the importance of chemokines in AD as therapeutic targets. Clinical trials for patients with AD are not sufficient compared to those for rheumatoid arthritis (1). However, as indicated here, some antibodies and antagonists showed clinical improvement in AD model mice, and some clinical trials are currently going on. Thus, chemokine-targeting therapy is a promising approach for future clinical application.
